# Aging Effect on Visuomotor Adaptation: Mediated by Cognitive Decline

**DOI:** 10.3389/fnagi.2021.742928

**Published:** 2021-10-28

**Authors:** Na Li, Guopeng Chen, Yong Xie, Zhongting Chen

**Affiliations:** ^1^Shanghai Key Laboratory of Brain Functional Genomics, Affiliated Mental Health Center, School of Psychology and Cognitive Science, East China Normal University, Shanghai, China; ^2^Shanghai Institute of Technical Physics, Chinese Academy of Sciences, Shanghai, China

**Keywords:** aging, visuomotor adaptation, age-related cognitive decline, motor planning, online motor control

## Abstract

The question of whether and how aging affects humans’ visuomotor adaptation remains controversial. This study investigates how the effect of aging on visuomotor adaptation is related to age-related cognitive declines. We compared the performance of 100 older people (age: 55–82 years) and 20 young adults (age: 18–27 years) on a visuomotor adaptation task and three cognition tasks. A decline in visuomotor adaptation of older people was well observed. However, this decline was not strongly correlated with chronological age increase but was associated to the age-related declines of cognitive functions and speed of motor planning. We then constructed a structural mediation model in which the declined cognitive resources mediated the effect of age increase on the decline in visuomotor adaptation. The data from the present study was well-explained by the mediation model. These findings indicate that the aging effect on visuomotor adaptation mainly reflects the age-related decline of cognitive functions, which results in insufficient explicit processing on visual perturbation during visuomotor control.

## Introduction

One fundamental function of humans’ sensorimotor system is to adapt to extrinsic and/or intrinsic environmental changes. This function of visuomotor adaptation has been extensively studied by examining rapid adaptation in simple reaching tasks with deviated visual feedback. With this approach, many studies have found evidence that healthy young people can rapidly adapt to visual feedback deviation (i.e., extrinsic environmental changes) in simple reaching tasks (e.g., Krakauer et al., 1999; Ghilardi et al., 2000; Taylor and Ivry, 2013; Galea et al., 2015; Haar et al., 2015).

While rapid sensorimotor adaptation has been observed for healthy young people, it is not yet clear how aging affects sensorimotor adaptation. Given that the functioning of cognitive systems (Raz et al., 2000) and sensorimotor performance (Cerella, 1990) substantially decline across the human lifespan, it is intuitive and reasonable to expect that visuomotor adaptation would decline with age increase as well. However, previous empirical studies have reported mixed findings. Multiple studies have reported that the reaching adaptation of older people could be subject to minor decline or even be well-preserved compared to that of young adults (e.g., Buch et al., 2003; Heuer and Hegele, 2008b; Cressman et al., 2010). On the other hand, multiple studies found that older adults had significantly weaker adaptation (i.e., smaller angular deviation against the direction of perturbation) or slower adaptation (i.e., more trials before angular deviation reaches the maximum) than young adults (e.g., Buch et al., 2003; Bock, 2005; Bock and Girgenrath, 2006; Seidler, 2006; Heuer and Hegele, 2008a).

Roller et al. (2002) tested 73 subjects (age: 20–80 years) by asking them to throw balls while wearing a set of prisms that displaced the visual scene by 20° to the right. The researchers did not observe systematic change in measures of visuomotor plasticity with advancing age. However, a number of studies using a reaching task with visual perturbation have found that older people performed significantly worse than young adults under a condition in which online feedback of angular deviation could be explicitly noticed or participants did open-loop control (Buch et al., 2003; Bock, 2005; Bock and Girgenrath, 2006; Seidler, 2006; Heuer and Hegele, 2008a, 2009, 2011, 2014; Hegele and Heuer, 2010a,b, 2013). However, Heuer and Hegele (2008b) reported no age-related decline of adaptation with a simplified version of the reaching task. Likewise, both Cressman et al. (2010) and Buch et al. (2003) found that older people could perform the visuomotor adaptation task as well as young adults. They both suggested that adults over 60 years old recalibrated their sensory and motor systems to preserve visuomotor adaptation.

Despite a number of studies that have investigated the correlation between age and the decline of visuomotor adaptation in reaching, only two recent studies used large samples of older people. Wolpe et al. (2020) recruited 183 older participants (age: 50–89 years) and Vandevoorde and de Xivry (2019) recruited 71 (age: 59–76 years). One possible reason for the mixed results from previous studies could be that aging effects on visuomotor adaptation vary among different age ranges and tasks. The present study further investigates the issue using a relatively large sample of older people whose ages were evenly distributed in a wide range (55–82 years), providing better conditions for data analyses.

Another possible reason for the mixed results could be individual differences in functions other than visuomotor control. Aging affects cognitive functions, and this effect varies largely across individuals (Rapp and Amaral, 1992; McClearn et al., 1997). Previous research has suggested that interindividual variabilities of cognitive function, including decision-making, inhibition, and flexibility as measured by a battery of reaction-time tests, and of adaptive visuomotor control were larger within older subjects than within young subjects (Bock and Girgenrath, 2006). Despite recent studies that conducted cognitive-test batteries prior to the visuomotor adaptation task to check whether older subjects had declined cognitive functions compared to young adults (Heuer and Hegele, 2008a,b, 2009, 2011, 2014; Hegele and Heuer, 2010a,b; Vandevoorde and de Xivry, 2019), how cognitive functions and visuomotor adaptation covary across the life span is yet to be further investigated.

In addition, it is still controversial whether visuomotor adaptation is linked to cognitive functions. Simon and Bock (2017) observed that the aging effects on adaptation were associated with the ability for inhibition, whereas an earlier study (Bock and Girgenrath, 2006) did not find an association between declined inhibition function and aging effects on the visuomotor adaptation of older people. More importantly, according to our knowledge, only one study (Vandevoorde and de Xivry, 2019) has investigated the associations between declined cognitive functions and aging effects on adaptive visuomotor control using a relatively large sample of older people. To address this issue, the present study used a large sample of 100 older participants whose cognitive functions were evaluated to investigate their relationships with visuomotor adaptation.

The present study also investigated how decline in visuomotor adaptation was related to visuomotor planning and online visuomotor control. Prior to a movement’s initiation, cognitive factors coupled with a visual “planning” representation together determine the following motor program (Glover, 2004). Initiation time (IT) refers to the duration of the planning phase, which included several sub-processes of target identification, response selection, and movement planning or reprogramming (Liu et al., 2008). For instance, when participants need to avoid collision with obstacles and reach a target, it would incur a larger reaction-time cost for movement preparation (Wong et al., 2016). Recent evidence has also shown that anticipatory motor planning proficiency rapidly declined around 70 years of age in an end-state comfort task (Scharoun et al., 2016; Stöckel et al., 2017; Wunsch et al., 2017). However, planning ability has not been sufficiently investigated for its role in the visuomotor adaptation across the life span. Therefore, this study tested the relationship between IT and visuomotor adaptation to investigate whether variation of visuomotor planning is related to visuomotor adaptation decline of older people.

Different from IT, movement time (MT) refers to an online control process that uses limited but quickly updated visual information to adjust movements during movement execution (Glover, 2004; Liu et al., 2008). Empirical evidence has suggested that online motor corrections are at least partially automatic. Correction of arm movement could be completed between 125 and 350 ms without awareness (Pisella et al., 2000; Johnson et al., 2002; Chen and Saunders, 2016). In this study, we used MT as a measure of the speed of online motor adjustment, so as to investigate the relationship between age-related changes in online motor control and the aging effect of visuomotor adaptation.

This study aimed to systematically investigate how aging effects on visuomotor adaptation vary across different age groups and the association between aging effects of visuomotor adaptation and the decline of cognitive functions, using a relatively large sample of older people (100 people) whose ages were evenly distributed in a wide range (56–82 years old). We conducted a typical rapid aiming task with 30°counterclockwise rotation (cursor relative to hand) perturbation, which was supposed to induce explicit visuomotor adaptation. 100 elderly people with a control group of 20 young people (age: 19–27 years) voluntarily participated in the study (see Table 1). We hypothesized that performance in visuomotor adaptation declined with aging and this decline was associated to worse performance on the cognitive tasks that measured inhibition control function, visual-spatial ability, and processing speed. We also analyzed whether the decline of visuomotor adaptation was associated to the age-related change of visuomotor planning and online visuomotor control, measured by the IT and MT of hand movement during the adaptation phase, respectively.

**TABLE 1:** Summary of participant demographics across age groups.

Age			*N*	Sex (male/female)	Education (years)

Range	*M*	*SD*			
18.8–27.1	21.4	2.4	20	10/10	15.5
55.9–59.8	58.0	1.4	20	10/10	10.2
60.7–64.8	62.7	1.4	20	10/10	10.9
65.3–69.9	67.4	1.7	20	10/10	10.5
70.1–74.8	72.3	1.5	20	10/10	11.2
75.2–82.3	78.3	2.2	20	10/10	11.4

## Materials and Methods

### Participants

One hundred and twenty participants in total volunteered for the present study. 100 healthy right-handed older participants were recruited and stratified by age and gender, resulting in 10 men and 10 women in each of five age brackets (55–59, 60–64, 65–69, 70–74, and 75+ years). Twenty additional healthy right-handed young participants (range: 18–28 years, mean: 21.4 years, and SD: 2.4 years, 10 women) were recruited as a control group. The handedness of the participants was checked using the Edinburgh Handedness Inventory (Oldfield, 1971). Visual acuity was measured binocularly for all participants to confirm normal or corrected-to-normal vision. No participant had a history of neurological diseases, psychiatric disorders, or musculoskeletal dysfunctions. All were paid 50 RMB for their participation. The study was approved by and conformed to the standards of the Human Research Ethics Committee for Non-Clinical Faculties at East China Normal University.

According to the existing literature, studies that investigated the differences of visuomotor adaptation between older and young people often used sample sizes around 20 people for each age group (e.g., Buch et al., 2003; Bock, 2005; Bock and Girgenrath, 2006; Seidler, 2006; Heuer and Hegele, 2008a,b, 2009, 2011, 2014; Cressman et al., 2010; Hegele and Heuer, 2010a,b). To match the designs and compare the results with the previous studies, we chose to recruit 20 people for each age group.

### Apparatus

**FIGURE 1:**
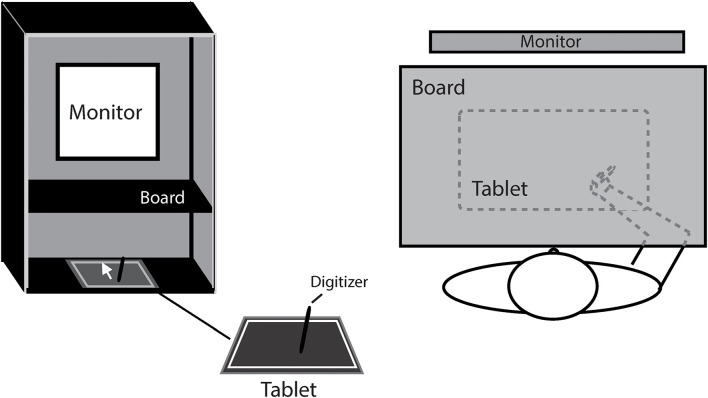
The illustration of experiment setting.

Figure 1 shows the experiment setup. The participant was seated comfortably in a dim room, facing an LCD monitor (NEC 192WG, 1440 × 768 pixels, 19-in., 60 Hz) which was positioned in the frontal plane 50 cm from the participants’ eyes. To block the participant from the visual feedback of hand movement, a board was positioned under the participant’s chin to make the right hand invisible. Hand movement trajectories were sampled at a rate of 40 Hz by a digitizer (216 × 135 mm, Wacom, Intuos). The experiment was programmed in MATLAB using the Psychtoolbox package (Brainard, 1997; Pelli, 1997; Kleiner et al., 2007).

### Tasks and Procedure

Each participant first passed a line drawing task, through which participants who had visuomotor deficits would be screened out. Then the participant performed a computer-based visuomotor adaptation task with a visual perturbation of 30° rotation and three paper-based tests for cognitive functions. Each participant spent about 30–50 min to finish the whole experiment.

#### Line Drawing

The line drawing task was adapted from the task by Alty et al. (2017). The target was placed on an invisible ring whose center was located on the center of the square white paper and whose radius was 100 mm. Target directions were displayed 0°, ±22.5°, ±45°, and 180° relative to the vertical midline of the ring. Participants held the pen to draw straight lines from the center to the targets in a clockwise sequence. Those who showed obvious hand tremors during the straight line drawings would be screened out and not continue to participate in the visuomotor adaptation tasks.

#### Visuomotor Adaptation Task

**FIGURE 2:**
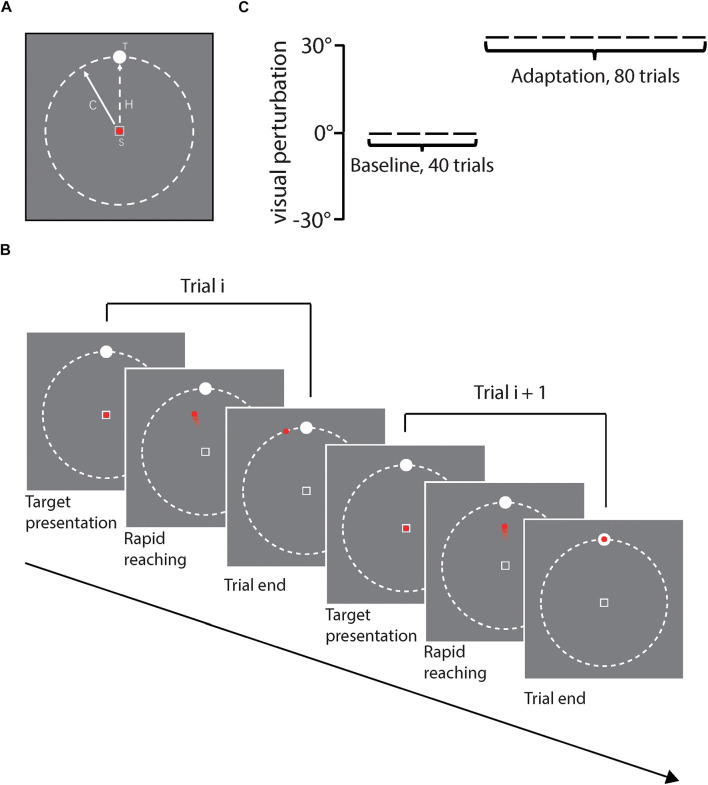
**(A)** Illustrates stimuli presented on each trial, including the start position (S) with the red point cursor inside, and white circular target (T). The solid line with arrow represents the cursor moving direction and the dash line represents the actual hand movement direction (H). **(B)** illustrates the task procedure of two sample trials, **(C)** illustrates the experiment design for each participant, including first 40 baseline trials and then 80 adaptation trials. Rotation direction perturbation was fixed to 30°counterclockwise in reference to the actual hand movement direction in the adaptation phase.

As shown in Figure 2A, stimuli were presented against a gray background. In each trial, a hollow square with white edges (1.2° × 1.2° of visual angle) was presented at the center of the screen as the start position. A red point (1° of visual angle) was presented to index the position of the cursor online and always in the center of the start square at the beginning of each trial. A white circular target (diameter: 1.4° of visual angle) was placed on an invisible ring whose center was located in the center of the screen and whose radius was 20.3° of visual angle. Target positions were 0°, ±22.5°, and ±45° relative to the vertical midline line of the ring, randomly chosen for each trial. Figure 2B illustrates the procedure of a certain trial in the task. At the beginning of each trial, the participant moved the digitizer to the center of the tablet and a red point appeared on the center of the screen. The target was presented at the same time. The participant controlled the red point using the digitizer by the right hand and was asked to move the red point to reach the visual target as quickly and accurately as possible. The red point was kept visible for online feedback during the movement (i.e., closed-loop control). Both the red point and the target disappeared immediately when the red point moved beyond the invisible ring boundary. After that, the participant moved the digitizer back to the start position. The next trial would not start until the participant located the digitizer onto the start position. The whole movement trajectory was tracked and recorded online by the digitizer during the movement.

As Figure 2C shows, each participant first finished 15 trials without perturbation for practice and then started the formal experiment, in which the participant sequentially received 40 baseline trials and 80 adaptation trials. For each trial, the target position was randomly chosen from 5 alternative positions (i.e., 0°, ±22.5°, ±45°), and trials with different target positions were collapsed for data analysis.

#### Tests of Cognitive Capabilities

We measured three cognitive capabilities (i.e., inhibition control function, visual-spatial ability, and processing speed) for each participant. We first administered a Chinese version of the Stroop Color and Word Test, which was translated from the original version of Stroop Color and Word Test one (Stroop, 1935), as a measure of the inhibition control function. In this test, the participant needed to name aloud the colors of colored rectangles (baseline conditions) or of color words that were printed in incongruent colors (inhibition conditions) in the different blocks. The participants were asked to respond as quickly and correctly as possible, and their reaction times were recorded manually. The inhibition control was measured by the mean RT difference between the correct trials in inhibition conditions and in baseline conditions for each participant.

The Block Design Test was from the Chinese version of Wechsler Adult Intelligence Scale (*abbr.* WAIS – RC; Gong, 1982). For the Block Design Test, the participant was presented with a set of blocks in different colors that were arranged into a specific pattern every time. The participant was asked to replicate the pattern in a limited time. The participant was tested with four blocks in the first 6 trials and then with nine blocks. The test would be terminated after three consecutive failures. This test was used to measure the individual’s visual-spatial ability.

The Digit Symbol Test was also from WAIS-RC. In the Digit Symbol Test, each of the digits (i.e., 1–9) was mapped onto a unique, easy-to-draw symbol (e.g., “o,” “=,” and “×”). According to the mapping rule (shown at the top of the answer sheet), the participant was asked to fill the corresponding symbols under each of a set of randomly selected digits. The participant needed to fill as many symbols as possible in 90 s. This test was used to measure the individuals’ processing speed. The experimenter would score the test by giving one point for each correct response.

For evaluation of cognitive functions, some of previous studies used more complete batteries of cognitive tests (e.g., Heuer and Hegele, 2008b, 2009; Hegele and Heuer, 2010a,b; Anguera et al., 2011). However, considering that a too long session might not be suitable for older participants, the present study only included three cognitive tests to maintain the duration of the whole experiment within 1 h for each participant. The three tests may not provide a most comprehensive evaluation of cognitive functions, but they together should be sufficient to give a reliable evaluation of a general cognitive function. The existing literature has shown that, despite multiple aspects of cognitive functions and various tests on those, individual differences of performances on most cognitive tests had moderate-to-high correlations with each other, indicating a common component that underlies performances on most cognitive tasks (e.g., McCabe et al., 2010; Huang et al., 2012; Kovacs and Conway, 2016; Shipstead et al., 2016). We also used structural equation modeling to construct a latent variable to represent a general cognitive resource so that the relationships among age, visuomotor adaptation and general cognitive functions could be further investigated (for details, see section *“Correlation Analyses of Visuomotor Adaptation, Kinematics, and Cognitive Functions”*).

### Data Analysis

The hand movement onset for each trial was marked when the velocity first exceeded 5% of the peak velocity of the hand movement in this trial. The movement orientation of each trial was defined by direction from the movement onset point to the point on which peak velocity was reached, so as to minimize the influence from movement corrections based on feedback. To calculate the angular deviation of hand movement in each trial, a standard vector was defined from the position of the start point to the target point, and the angular deviation in this trial was defined as the orientation difference between the movement orientation and the orientation of the standard vector.

We measured the adaptation effect of hand movement by the mean angular deviations of hand movement from the last 15 adaptation trials minus the mean deviation from the baseline trials. This manipulation helped to remove the variation of individual biases in hand movement from the adaptation effects (Leow et al., 2012; McDougle et al., 2015; Song and Smiley-Oyen, 2017).

In addition, we analyzed the kinematics of the hand movements, including the IT of hand movement, which was defined by the time from stimulus presentation to the onset of movement for each trial, and the MT of hand movement, which was defined by the time from the onset of movement to when the cursor reached the invisible boundary for each trial.

## Results

### Aging Effects on Visuomotor Adaptation

We first analyzed the mean angular deviations in the baseline phase of the reaching task to investigate whether the older and young participants had differences in constant biases in the reaching task. An ANOVA showed that they had no biases difference in the baseline trials [*F* (1,118) = 0.793, *p* = 0.375, and partial η^2^ = 0.007], and both showed a small clockwise bias, namely, young adults (−1.54° ± 1.44°) and older adults (−1.13° ± 1.96°). This effect might be because all the participants were right-handed. Then, we compared the mean standard deviations of individuals’ angular deviations in the baseline phase between two groups to test whether two groups differed in reaching precision, and found that the older participants had a significantly higher mean standard deviation [*F* (1,118) = 33.06, *p* < 0.001, and partial η^2^ = 0.219], indicating that the visuomotor control of the older participants were less accurate than of the young participants.

**FIGURE 3:**
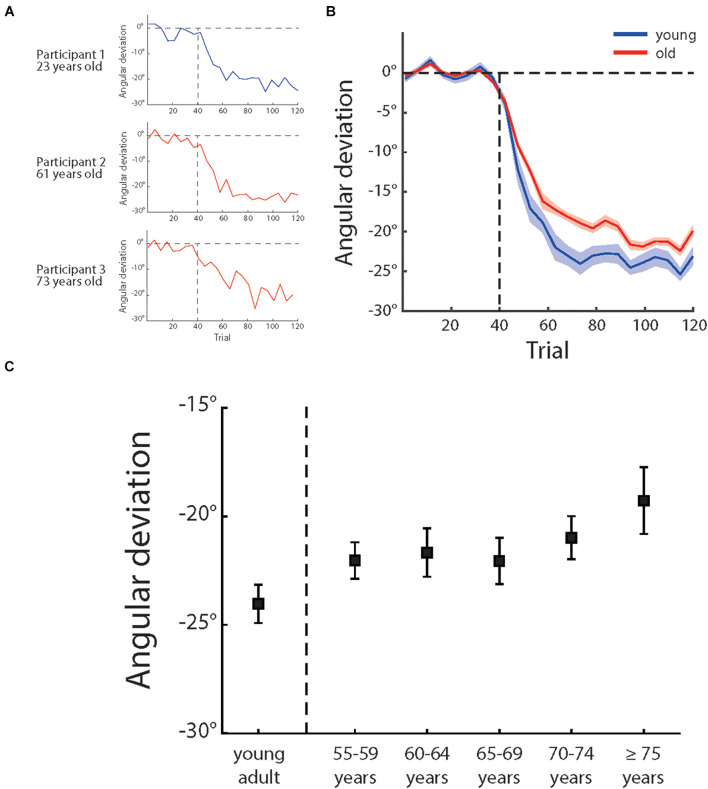
**(A)** Illustrates the angular deviation as a function of trial number of three sample participants of different age groups and **(B)** illustrates the mean angular deviation as a function of trial number of young adults (blue) and older people (red). The shadow areas depict ± 1 standard errors, **(C)** illustrates the mean angular deviation of last 15 adaptation trials of different age groups (for each group, *n* = 20). The error bars depict ± 1 standard errors.

Over the adaptation period, the cursor moving direction was rotated by 30°CCW. A best solution to counter cursor rotation was to adjust the hand movement direction by 30°clockwise with reference to the direction from the start point to the target point. Figure 3A illustrates how angular deviation varied across trials for three sample participants and Figure 3B shows the mean angular deviations of the young participants and the older participants across the experiment. One can see that, although both the young and older sample participants showed adaptation to compromise visual perturbation, the adaptation of the older participants were not as sufficient as of the young participants. The young adults group had a mean deviation of −24.04° (±0.88° s.e.) and older adults group −21.21° (±0.51° s.e.) in the last 15 trials (also see Figure 3C). An ANOVA showed a significant main effect of age groups, *F* (1,118) = 5.47, *p* = 0.021, and partial η^2^ = 0.044, confirming more visuomotor adaptation of the young participants.

**FIGURE 4:**
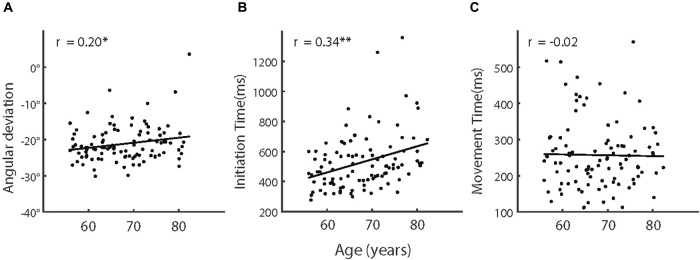
**(A)** Adaptive angular deviation, **(B)** initiation time, and **(C)** movement time as a function of age of older participants. The solid lines indicate linear regression model fits and rs represent the Pearson correlations between variables. Note, ^∗^*p* < 0.05; ^∗∗^*p* < 0.005.

We then analyzed the adaptation effect among the five age groups (i.e., 55–60 years, 60–65 years, 65–70 years, 70–75 years, and over 75 years) of the older participants (see Table 1 for more information of the groups). However, an ANOVA showed that the effect of age groups was not significant, *F* (4,95) = 0.881, *p* = 0.478, and partial η^2^ = 0.036. We also calculated the correlation between age and adaptation and found a weak but significant correlation, *r* (98) = 0.20, *p* = 0.044 (see also Figure 4A). These results indicated that, despite the aging effect on visuomotor adaptation, the relationship between the increase of age and the decline of visuomotor adaptation was not strong within older participants.

### Aging Effects on Kinematics: Initiation Time and Movement Time

To analyze the IT and MT differences between the young and older participants during the baseline phase and the adaptation phase (last 15 trials), we first performed two 2 (phase) × 2 (young vs. older) mixed-design ANOVAs for mean IT and MT, respectively. The main effects of phase were significant for both IT and MT [IT: *F* (1,118) = 8.95, *p* = 0.003, and partial η^2^ = 0.071; MT: *F* (1,118) = 11.90, *p* = 0.001, and partial η^2^ = 0.092], indicating longer IT and MT in the adaptation phase than in the baseline phase. For IT, we also found a significant main effect of young vs. older [*F* (1,118) = 29.76, *p* < 0.001, and partial η^2^ = 0.201], indicating longer IT of the older participants. However, the main effect of young vs. older was not significant for MT, *F* (1,118) = 0.187, *p* = 0.666, and partial η^2^ = 0.002. The interaction between two factors was not significant for IT [*F* (1,118) = 1.897, *p* = 0.173, and partial η^2^ = 0.016] and significant for MT [*F* (1,118) = 4.63, *p* = 0.034, and partial η^2^ = 0.038]. The significant interaction for MT indicated that the MT difference between the phases was larger for the older participants.

**FIGURE 5:**
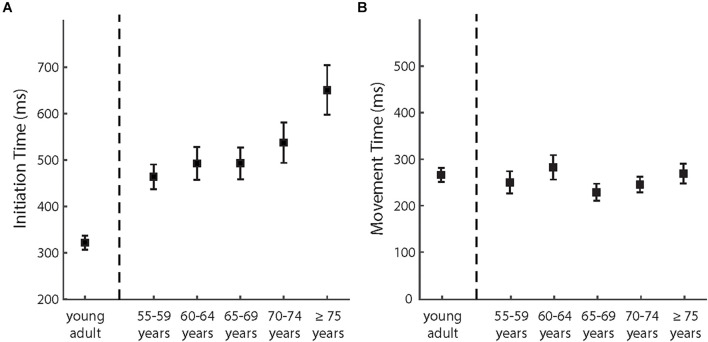
**(A)** The mean initiation times and **(B)** the mean movement times of different age groups in the adaptation phase. The error bars depict ± 1 standard errors.

We then analyzed how IT and MT in the adaptation phases varied among the age groups of the older participants (see also Figure 5). The effect of age groups was significant for IT [*F* (4,95) = 3.45, *p* = 0.011, and partial η^2^ = 0.127] but not for MT [*F* (4,95) = 0.937, *p* = 0.446, and partial η^2^ = 0.038]. As shown in Figure 4B, IT increased with advancing age across the groups. The correlation analyses provided consistent results. The correlation was significant between age and IT [*r* (98) = 0.34, *p* = 0.001] but not significant between age and MT [*r* (98) = −0.02, also see Figure 4C].

### Aging Effects on Cognitive Capabilities

**FIGURE 6:**
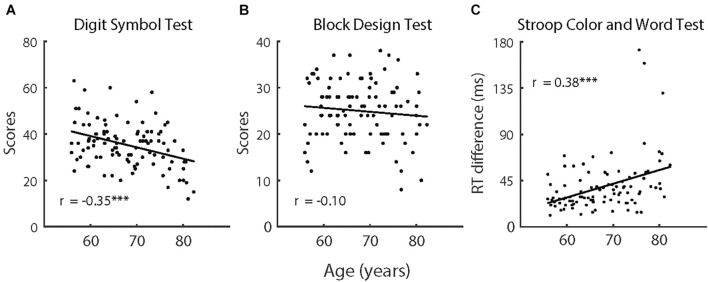
**(A)** The scores in Digit Symbol Test, **(B)** the scores in Block Design Test, and **(C)** the reaction time difference in Stroop Color and Word Test as a function of age of older participants (*n* = 100). The solid lines indicate linear regression model fits and rs represent the Pearson correlations between variables. Note, ^∗∗∗^*p* < 0.001.

The comparisons of cognitive capabilities showed that the older participants had significantly poorer performance on all three tasks (i.e., Digit Symbol Test, Block Design Test, and Stroop Color and Word Test), *F* (1,118) > 15.64, *p* < 0.001, and partial η^2^ = 0.130. As depicted in Figure 6, we also analyzed trends in cognitive functions with age for the older participants. Considering that there were large differences in age between the older participants and the control group, the control group was not included in the correlation analyses that were directly related to age. Performance on the Digit Symbol Test and Stroop Color and Word Test both had significant correlations with age [Digit Symbol Test: *r* (98) = −0.35, *p* < 0.001; Stroop Color and Word Test: *r* (98) = 0.38, *p* < 0.001], whereas the correlation between performance on the Block Design Test and age was not significant [*r* (98) = −0.10, *p* = 0.333].

### Correlation Analyses of Visuomotor Adaptation, Kinematics, and Cognitive Functions

**FIGURE 7:**
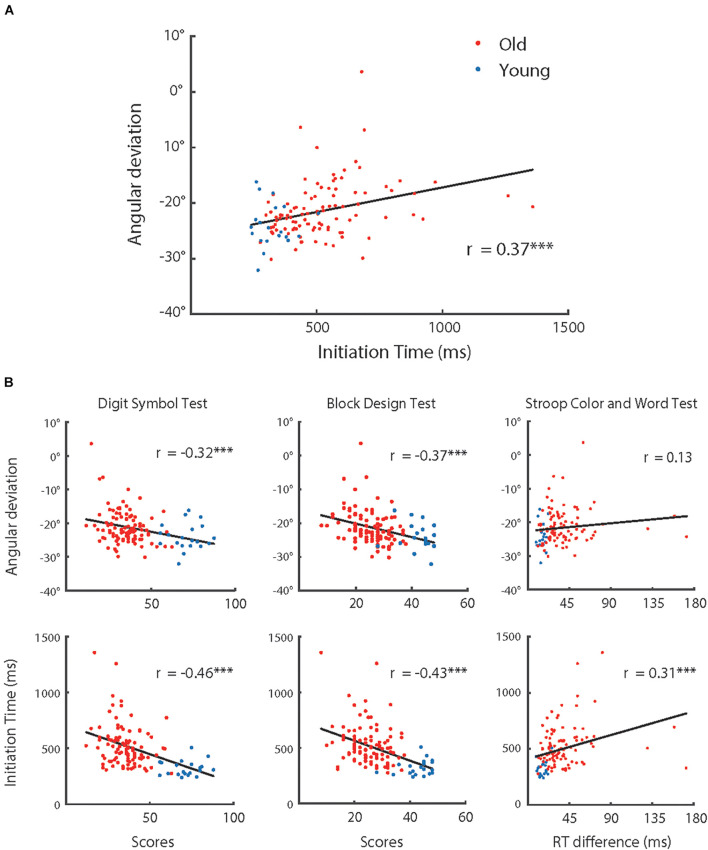
**(A)** Adaptive angular deviation as a function of initiation time of hand movement, **(B)** adaptive angular deviation (upper row) and initiation time of hand movement (lower row) as functions of performance in cognitive tests (left: Digital Symbol Test; middle: Block Design Test; and right: Stroop Color and Word Test), respectively. The red dots represent the group of older participants (*n* = 100) and the blue dots represent the group of younger participants (*n* = 20). The solid lines indicate linear regression model fits and rs represent the Pearson correlations between variables. Note, ^∗∗∗^*p* < 0.001.

As shown in Figures 3, 4, the adaptive angular deviation and IT had a similar tendency for participants over 55 years old. Therefore, we conducted an analysis of correlation between them for all participants, which revealed that the correlation between angular deviation and IT was significant [*r* (118) = 0.33, *p* < 0.001]. We also tested whether the adaptive angular deviation and IT were correlated with performance on three cognitive tests (see Figure 7), respectively. For angular deviation, it was significantly related to scores of the Digit Symbol Test [*r* (118) = −0.32, *p* < 0.001] and the Block Design Test [*r* (118) = −0.37, *p* < 0.001] but not to scores of the Stroop Color and Word Test [*r* (118) = 0.13, *p* = 0.167]. The insignificant correlation between angular deviation and scores of the Stroop Color and Word Test might be due to that individual performances in this task were measured by differences between reaction times in different conditions. The process of finding differences might have resulted in the increase of measurement noise. For IT, all the correlations were significant [*r* (118) > 0.31, *p* < 0.001]. For the results of other correlation analyses, please see Table 2. Note that the correlation between MT and angular deviation was negative and statistically significant, indicating that the participants whose hand movements were faster showed more visuomotor adaptation. However, MT did not differ systematically with the participants’ age. Thus, the MT-adaptation correlation was apparently not related with the aging effect on visuomotor and was not further analyzed.

**TABLE 2:** Correlations between visuomotor adaptation, kinematics, and cognitive functions.

	Age	Angular deviation	IT	MT	Digit symbol test	Block design test
Age	–					
Angular deviation	0.26**	–				
IT	0.49***	0.33***	–			
MT	–0.04	−0.19*	−0.22*	–		
Digit symbol test	−0.83***	−0.32***	−0.46***	–0.02	–	
Block design test	−0.71***	−0.37***	−0.43***	0.02	0.77***	–
Stroop color and word test	0.45***	0.13	0.31***	0.13	−0.50***	−0.48***

Note: *p < 0.05; **p < 0.01; and ***p < 0.001.

The correlation analyses showed clear associations among performance on the cognitive tests and motor planning speed (i.e., IT) and similar aging effects on them. These findings together indicated that a common cognitive resource affected performance on both cognitive tests and motor planning and declined with increase of age. Moreover, the observed associations among performance on the cognitive tests, IT, and adaptive angular deviation indicated the possibility that there was an association between adaptive angular deviation and this hypothetical cognitive resource. Considering the relatively weak correlation between age and adaptation effect, we think that chronological age increase might not directly result in a decline of visuomotor adaptation, but this cognitive resource possibility mediated the relationship between age and adaptive angular deviation.

**FIGURE 8:**
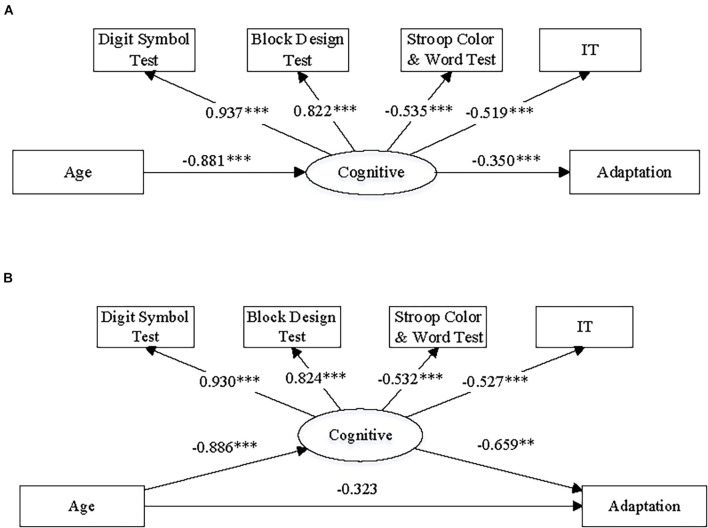
The diagrams illustrate the structures and summary results of **(A)** the full mediation structural equation model and **(B)** the partial mediation structural equation model, respectively. The numbers on the paths of the diagrams refers to standardized coefficients. Note, (1) “Cognitive” refers to cognitive resources; “Adaptation” refers to adaptive angular deviation; “IT” refers to initiation time of hand movement; (2) ^∗∗^*p* < 0.005 and ^∗∗∗^*p* < 0.001.

According to the cross-sectional design of the present study, we could not directly test causal relationship among chronological age increase, changes in visuomotor adaptation and cognitive function. However, to further test this possible mediation effect using a statistical approach, we did path analysis by constructing and fitting two structural equation models and using the data of all 120 participants. The structure of the models is illustrated in Figures 8A,B. We hypothesized a latent variable, labeled as “cognitive resource,” and included it in both models as a mediator between aging and adaptation effect. Using the methods of path analysis and structural equation modeling, we could test (1) whether there was a general cognitive resource that affected both performance on the cognitive tests and motor planning/initiation (i.e., IT) and (2) whether this cognitive resource mediated the effect from age to adaptive angular deviation from a perspective of statistics. In addition, we could test whether there was a full or partial mediation effect by comparing models with/without a direct path between age and adaptive angular deviation (Figures 8A,B).

The model construction and fitting were implemented by Mplus 7 (Muthén and Muthén, 1998-2017). The results of model fitting and the indices of goodness-of-fit are presented in Figure 8 and Table 3. The numbers on the diagrams refer to standardized coefficients of the paths. The indices of goodness of it were chosen according to a common practice (Schermelleh-Engel et al., 2003; Marsh et al., 2005). We also list the criteria for acceptable fit in Table 3. The full mediation model (Figure 8A) appeared to be acceptable based on the indices of goodness-of-fit, and both the paths related to mediation effect were significant, indicating that there was a significant mediation effect. On the other hand, the indices of goodness-of-fit indicated that the partial mediation model (Figure 8B) was acceptable as well. However, the direct path from age to adaptive angular deviation was not significant, indicating that the full mediation model was preferred. The full mediation effect suggested that aging did not directly result in decline of visuomotor adaptation, but the aging effect on visuomotor adaptation was more likely to be due to the age-related cognitive resource decline.

**TABLE 3:** Model fit indices for latent variable mediation model.

Model	χ ^2^	*df*	CFI (≥0.95)	TLI (≥0.95)	SRMR (≤0.10)	RMSEA (≤0.08)
Full mediation	14.558	9	0.984	0.973	0.040	0.072
Partial mediation	12.495	8	0.987	0.975	0.037	0.068

*Note: the ranges in the brackets refer to criteria of acceptable fit on indices of goodness-of-fit.*

## Discussion

The present study is focused on how aging affects visuomotor adaptation. In brief, we found that the older participants (>55 years) had less adaptation to visual feedback perturbation in a reaching task, compared to the young adults. However, this aging effect on visuomotor adaptation was not strongly associated to the chronological age of the older participants; instead, we observed that the effect was more correlated with the decline of performance on the cognitive tests and speed of motor planning. We then tested a structural mediation model, in which we hypothesized a latent variable, entitled “cognitive resource,” both accounted for performance on the cognitive tests and speed of motor planning and mediated the effect from chronological age to adaptive angular deviation. The fitting results confirmed this model and indicated that there was a full mediation effect of cognitive resource.

### Aging and Visuomotor Adaptation

Multiple studies have reported that visuomotor adaptation, as an important function of the visuomotor feedback system, declines in older people compared to young adults (Buch et al., 2003; Bock, 2005; Bock and Girgenrath, 2006; Seidler, 2006; Heuer and Hegele, 2008a, 2009, 2011, 2014; Hegele and Heuer, 2010a,b, 2013). From this study, the data consistently showed that the older group had less adaptive angular deviation by visual feedback perturbation. With a relatively large sample, we further tested whether this aging effect was correlated with chronological age. Not much literature has addressed this issue. In a very recent study, Wolpe et al. (2020) found a considerable correlation of 0.35 between age and adaptive angular deviation, which appears to be higher than observed in the present study (0.20). This difference could be caused by the different age ranges of the participants in the two studies. Wolpe et al. tested the correlation across the span of adulthood (age: 18–89 years) while we focus on older people (age: 55–82 years). As shown in Figure 2B of Wolpe et al., older people apparently had a larger variation in adaptive angular deviation than young adults had, which could possibly have caused a lower correlation within the older group. We also tested the correlation between age and adaptive angular deviation using all the participants in this study (though this analysis apparently violated the assumption of normal distribution of sample), and found a slightly higher correlation [*r* (118) = 0.26, *p* = 0.004]. But considering the sample distribution, we don’t think that any conclusion could be given with such a small difference. Another recent study also showed an insignificant correlation between age and visuomotor adaptation within older people (Vandevoorde and de Xivry, 2020).

Despite the observed weak correlation between chronological age and adaptive angular deviation, we found that adaptive angular deviation was also correlated with performance in cognitive tests, which was associated to adaptive angular deviation. These observed associations point toward the mediation effect of age-related cognitive function decline on the aging effect of visuomotor adaptation. The mediation model (Figure 8A) confirms this effect. Multiple previous studies have shown age-related cognitive function decline (Salthouse, 1996; Raz et al., 2000; Park et al., 2003). Recently, some studies have further noted that this age-related decline possibly contributed to the aging effect on visuomotor adaptation. For example, Vandevoorde and de Xivry (2019, 2020) found that age-related visuomotor adaptation decline was likely to link to the decline of working memory capacity by aging. Wolpe et al. (2020) also reported that aging effect on visuomotor adaptation was related to short-term memory decline. In an early study, Heuer and Hegele (2008a) did not find the correlations among age, visuomotor adaptation, and cognitive functions but still pointed out that both performance on the Digit Symbol Test and visuomotor adaptation declined with the increase of age. In addition, this study found that speed of visuomotor planning (i.e., IT) was related to the aging effect on visuomotor adaptation. The present finding was consistent with previous studies (Scharoun et al., 2016; Stöckel et al., 2017; Wunsch et al., 2017), which reported age-related decline in anticipatory motor planning.

The findings of this study can potentially explain why the aging effect on visuomotor adaptation was not observed in some studies. For instance, in Heuer and Hegele’s (2008b) study, older people did not show a decline of visuomotor adaptation in a simplified visuomotor adaptation task. This could be because the simplification of the task decreased the involvement of explicit visuomotor planning, making the decline of cognitive resources less influential in the task performance. Similarly, in Buch et al. (2003) and Cressman et al. (2010), the researchers gradually changed the visuomotor feedback and further provided proprioceptive feedback, both of which were likely to reduce the involvement of explicit visuomotor planning and potentially helped older people to perform as well as young adults. These previous findings are consistent with our conclusion that the decline of cognitive resources is the main cause of the aging effect on visuomotor adaptation.

Based on the findings that performance on the cognitive tests and speed of motor planning were correlated with both chronological age and adaptive angular deviation, we propose a mediation model (Figure 8A), in which age-related decline of cognitive resources underlies the aging effect on visuomotor adaptation. The indices of goodness of fit show that the mediation model statistically explains the observed data well (see Table 3). In the next subsection, we further discuss the relationships among visuomotor adaptation, motor planning, and cognitive functions from a more general perspective.

### Visuomotor Adaptation, Motor Planning, and Cognitive Functions

The results of the present study have demonstrated that the aging effect on visuomotor adaptation was mediated by age-related cognitive function decline. An explanation of these findings is that cognitive resources are shared for cognitive functions (e.g., working memory, spatial representation, and numerical representation), motor planning, and visuomotor adaptation (especially explicit visuomotor adaptation). This explanation is supported not only by evidence from research on older people but also by evidence from young adults and children. For example, studies on young adults have shown that both working memory capacity (Christou et al., 2016; Wolpe et al., 2020) and performance intelligence quotient (Anwar et al., 2015) could predict explicit visuomotor adaptation. Cognitive cost by dual task (Vandevoorde and de Xivry, 2020) and attentional disorders (Kurdziel et al., 2015) both affected visuomotor adaptation as well. These findings convergently indicated shared resources for visuomotor adaptation and cognitive functions. Moreover, Logan and Fischman (2011) reported that motor planning interfered with performance in memory recall, and Pennequin et al.’s (2010) research on child development found covariation between motor planning and executive functions, suggesting that motor planning and cognitive functions have shared resources.

Heuninckx et al. (2005, 2008) pointed out that aging results in more cognitive demand and increased neural recruitment during motor tasks. Vandevoorde and de Xivry (2020) further proposed a shared cognitive resource hypothesis to explain the aging effect on explicit visuomotor adaptation. This hypothesis is consistent with the findings of the present study that performance in cognitive functions and speed of motor planning predicted adaptive angular deviation better than chronological age. The mediation model tested in the present study also supports Vandevoorde and de Xivry’s (2020) hypothesis by identifying shared cognitive resources change as a full mediator from chronological age increase to visuomotor adaptation decline. In other words, the findings of the present study, with the previous findings, indicate that the aging effect on visuomotor is not an independent effect but reflects the age-related decline of cognitive functions. Visuomotor adaptation at least partially relies on the same cognitive mechanisms that underlie a set of cognitive functions (e.g., working memory, spatial representations) and motor planning.

Recent evidence from neuroimaging studies also supports this explanation. Anguera et al. (2010) found that individual differences in visuomotor adaptation performance could be partially attributed to differences in spatial working memory and these two tasks activated overlapping brain regions, including the right dorsolateral prefrontal cortex and bilateral inferior parietal lobules. In a follow-up study (Anguera et al., 2011), they further found that reduced activation of these regions contributed to worse performances in both spatial working memory and visuomotor adaptation tasks. Wolpe et al. (2020) reported that the observed decline of visuomotor adaptation by aging was associated to the structural retention of the medial temporal lobe, which is related to cognitive functions such working memory and attention. In contrast, they did not observe an association between decline of visuomotor adaptation and retention of cerebellum, which involves in the internal model recalibration of motor control. All these results are consistent with the findings of the present study that aging effect on visuomotor adaptation was directly due to the age-related decline of cognitive functions, and support Vandevoorde and de Xivry (2019)’s argument that impaired internal model recalibration of motor control is not the main inducement of the age-related decline of visuomotor adaptation.

Despite that the shared cognitive resource hypothesis well explained the relationship between explicit visuomotor adaptation and cognitive functions, it apparently could not predict how aging affects implicit visuomotor adaptation. Previous studies have reported that explicit visuomotor adaptation was affected by aging, whereas implicit visuomotor adaptation was not (Bock and Girgenrath, 2006; Heuer and Hegele, 2009, 2011; Hegele and Heuer, 2010a,b, 2013; Huang et al., 2017). In the present study, we used a sudden rotation perturbation of 30°CCW, which could be easily noticed by participants. So, although we did not separately measure the explicit and implicit components, the participants apparently introduced explicit strategies for visuomotor adaptation. As mentioned above, explicit visuomotor adaptation is associated to cognitive functions, and from this perspective, our findings are generally consistent with the existing literature. However, we could not distinguish the contributions of the explicit and implicit components on the aging effect on visuomotor adaptation with the design. Note that a rotation perturbation of ≥30° is not a necessary condition for either explicit adaptation or detecting an age-related effect on adaptation. In both Buch et al. (2003) and Cressman et al. (2010), the final perturbation magnitude was equal to or larger than 30°. However, the perturbations were both gradually increased from zero to maximum, and neither study found a significant effect of aging on adaptation. So we consider that aging effect on adaptation is dependent on not only magnitude of perturbation but how perturbation is presented as well, which shall be further investigated in future research.

Compared to speed of motor planning (i.e., IT of hand movement), MT did not differ between young adults and older people in the present study. This was possibly because MT is more associated with online visuomotor control, which is generally considered as an automatic process (Pisella et al., 2000; Johnson et al., 2002; Chen and Saunders, 2016) and does not apparently involve explicit strategies. This also explains why MT was not significantly correlated with performance in cognitive functions. Although MT was negatively correlated with adaptive angular deviation, it was more likely to be due to individual differences in online visuomotor control than to age-related variation (see Table 2).

## Conclusion

This study found an age-related decline of visuomotor adaptation that was mediated by performance in cognitive tests and speed of motor planning of individual participants. We proposed a structural model with a latent variable, entitled “cognitive resource,” which was due to both performance in cognitive tests and speed of motor planning and mediated the association from aging to visuomotor adaptation decline. These findings are consistent and extend the existing literature on visuomotor adaptation and aging.

## Data Availability Statement

The datasets presented in this study can be found in online repositories. The names of the repository/repositories and accession number(s) can be found below: https://osf.io/x2jnf/.

## Ethics Statement

The studies involving human participants were reviewed and approved by Committee on Human Research Protection, East China Normal University. The patients/participants provided their written informed consent to participate in this study.

## Author Contributions

NL and ZC developed the conceptual framework, conceived and designed the experiments. NL and YX programmed and performed the experiments. NL, YX, and ZC analyzed the data. NL, GC, and ZC wrote the manuscript. All authors contributed to the article and approved the submitted version.

## Conflict of Interest

The authors declare that the research was conducted in the absence of any commercial or financial relationships that could be construed as a potential conflict of interest.

## Publisher’s Note

All claims expressed in this article are solely those of the authors and do not necessarily represent those of their affiliated organizations, or those of the publisher, the editors and the reviewers. Any product that may be evaluated in this article, or claim that may be made by its manufacturer, is not guaranteed or endorsed by the publisher.
